# Neither Beetroot Juice Supplementation nor Increased Carbohydrate Oxidation Enhance Economy of Prolonged Exercise in Elite Race Walkers

**DOI:** 10.3390/nu13082767

**Published:** 2021-08-12

**Authors:** Louise M. Burke, Rebecca Hall, Ida A. Heikura, Megan L. Ross, Nicolin Tee, Georgina L. Kent, Jamie Whitfield, Sara F. Forbes, Avish P. Sharma, Andrew M. Jones, Peter Peeling, Jamie R. Blackwell, Iñigo Mujika, Karen Mackay, Marta Kozior, Brent Vallance, Alannah K. A. McKay

**Affiliations:** 1 Exercise and Nutrition Research Program, Mary MacKillop Institute for Health Research, Australian Catholic University, Melbourne, VIC 3000, Australia; Rebecca.hall@acu.edu.au (R.H.); ida.heikura@gmail.com (I.A.H.); meg.ross@acu.edu.au (M.L.R.); nicolin.tee@acu.edu.au (N.T.); Jamie.whitfield@acu.edu.au (J.W.); brent.vallance@athletics.org.au (B.V.); 2 Australian Institute of Sport, Bruce, Canberra, ACT 2616, Australia; Georgina.kent@ausport.gov.au (G.L.K.); sara.forbes@unisa.edu.au (S.F.F.); avish.sharma@triathlon.org.au (A.P.S.); Alannah.mckay@acu.edu.au (A.K.A.M.); 3 UniSA Online, University of South Australia, Adelaide, SA 5000, Australia; 4 Triathlon Australia, Burleigh Heads, Gold Coast, QLD 4220, Australia; 5 Sport and Health Sciences, University of Exeter, Heavitree Road, Exeter EX1 2LU, UK; A.M.Jones@exeter.ac.uk (A.M.J.); J.R.Blackwell@exeter.ac.uk (J.R.B.); 6 School of Human Sciences (Exercise and Sport Science), University of Western Australia, Crawley, WA 6009, Australia; peter.peeling@uwa.edu.au; 7 West Australian Institute of Sport, Mt Claremont, Nedlands, WA 6010, Australia; 8 Department of Physiology, Faculty of Medicine and Nursing, University of the Basque Country, 48940 Leioa, Basque Country, Spain; inigo.mujika@inigomujika.com; 9 Exercise Science Laboratory, School of Kinesiology, Faculty of Medicine, Universidad Finis Terrae, Santiago 7501015, Chile; karen.mackayp@gmail.com; 10 School of Exercise & Nutrition Sciences, Queensland University of Technology, Brisbane, QLD 4059, Australia; 11 Department of Physical Education & Sport Sciences, University of Limerick, V94 T9PX Limerick, Ireland; marta.kozior@ul.ie; 12 Athletics Australia, South Melbourne, Melbourne, VIC 3205, Australia

**Keywords:** exercise fuel, gut training, exogenous CHO, CHO loading, endurance sport, sucralose

## Abstract

Given the importance of exercise economy to endurance performance, we implemented two strategies purported to reduce the oxygen cost of exercise within a 4 week training camp in 21 elite male race walkers. Fourteen athletes undertook a crossover investigation with beetroot juice (BRJ) or placebo (PLA) [2 d preload, 2 h pre-exercise + 35 min during exercise] during a 26 km race walking at speeds simulating competitive events. Separately, 19 athletes undertook a parallel group investigation of a multi-pronged strategy (MAX; *n* = 9) involving chronic (2 w high carbohydrate [CHO] diet + gut training) and acute (CHO loading + 90 g/h CHO during exercise) strategies to promote endogenous and exogenous CHO availability, compared with strategies reflecting lower ranges of current guidelines (CON; *n* = 10). There were no differences between BRJ and PLA trials for rates of CHO (*p* = 0.203) or fat (*p* = 0.818) oxidation or oxygen consumption (*p* = 0.090). Compared with CON, MAX was associated with higher rates of CHO oxidation during exercise, with increased exogenous CHO use (CON; peak = ~0.45 g/min; MAX: peak = ~1.45 g/min, *p* < 0.001). High rates of exogenous CHO use were achieved prior to gut training, without further improvement, suggesting that elite athletes already optimise intestinal CHO absorption via habitual practices. No differences in exercise economy were detected despite small differences in substrate use. Future studies should investigate the impact of these strategies on sub-elite athletes’ economy as well as the performance effects in elite groups.

## 1. Introduction

Success in endurance events is underpinned by sustained or periodic achievement of high speed/power outputs which represent high relative and absolute exercise intensities, and are linked to an interaction between the athlete’s maximal aerobic power ($\dot{V}$O_2peak_), the fraction of $\dot{V}$O_2peak_ that can be sustained for the event distance, and the oxygen (O_2_) cost of movement (e.g., running/walking economy) [1,2,3,4,5]. Training and nutrition strategies for endurance performance aim to enhance various aspects of these characteristics [6], including ensuring that suitable substrates are able to fuel the event over its entire duration [4,7]. The economy of running or walking represents the relationship between oxygen utilisation and speed of locomotion [8], with a higher economy (lower oxygen cost for a given speed) at event-specific speeds being a better predictor of performance among a group of sub-elite/elite runners than $\dot{V}$O_2peak_ [9]. A range of training, environmental and biomechanical factors are known to affect running economy [10] and the recent breaking of the two-hour marathon barrier has renewed interest in economy as a determinant of endurance performance [11]. Indeed, testimonials [12] and studies related to new designs of running shoes [13] and the design of pacemaker formations to minimise air resistance for a targeted runner [14,15] have contributed to the achievement of this feat via the enhancement of running economy.

Several nutritional factors are also known to affect the economy of locomotion. Indeed, the popularity of beetroot juice supplements, used by athletes as a source of dietary nitrate, stems from early observations of a reduction in the oxygen cost of submaximal cycling [16,17] and running [18] following nitrate supplementation. Dietary nitrate can be serially reduced to nitric oxide (NO), providing a complementary pathway to the nitric oxide synthase (NOS-)supported conversion of arginine to NO [19]. The nitrate-NO pathway is of particular importance under the hypoxic and acidic conditions often occurring locally in the exercising muscle. In addition to enhancing oxygen delivery to the muscle or to specific fibres, nitrate supplementation is thought to reduce the oxygen cost of exercise via enhanced calcium handling and contractile efficiency [19], while earlier suggestions of enhanced mitochondrial efficiency are now disputed [20,21]. Although nitrate supplementation is more likely to benefit higher-intensity events [19], specific benefits to longer endurance events might occur via an attenuation of the gradual rise in oxygen cost during prolonged exercise [22] or enhanced $\;\dot{V}$O_2_ kinetics in the transition to a higher speed [23] as occurs when athletes change pace at critical times within a race. Previous studies of nitrate supplementation during prolonged exercise may have failed to detect benefits if the typical protocol of nitrate ingestion ~2 h pre-event was associated with gradual reduction in plasma nitrite concentrations before the end of the protocol [19]. Hence, supplementation protocols for endurance events might warrant nitrate intake before and *during* the event to maintain elevated nitrite concentrations [22]. 

Differences in exercise economy due to the choice of muscle substrate have also received recent attention following consistent findings from studies from our group [24,25,26,27] and others [28] that the substantial increase in muscle fat oxidation achieved by adaptation to a ketogenic low-carbohydrate (CHO), high-fat diet is associated with an increase in the oxygen cost of exercise at speeds which are relevant to the race performance of elite endurance athletes [29]. Indeed, as demonstrated more than a century ago [30,31], and explained by the stoichiometry of oxidative reactions [32], CHO oxidation produces 5–8% higher energy yield per litre of oxygen consumed through oxidative phosphorylation. Although contemporary sports nutrition guidelines for endurance performance already promote strategies to match CHO availability to the fuel demands of the event [7,33], it is worth considering whether further, even subtle, increases in CHO utilisation during the event might enhance economy in a meaningful way; either allowing the athlete to increase their speed for the same oxygen utilisation or reducing the oxygen and metabolic cost of a given speed. For example, according to our modelling [4], a 55 kg marathon runner with a sustainable $\dot{V}$O_2_ of 3.75 L/min and energy cost of 180 mL/kg/km would achieve a sustainable marathon running speed of 20.83 km/hr, with a finishing time of 2:01:33. In this scenario, a 0.05 increase in respiratory quotient (e.g., from 0.85 to 0.90) would induce a ~0.9% increase in the energy liberated per L of oxygen consumed, translating into a similar improvement in running speed (to 21.02 km/hr) and a 66 s improvement in finishing time (2:00:27). Whether theoretical calculations like these translate into real-life performance improvements, and whether CHO availability/utilisation can be further increased above the rates already achieved by elite endurance athletes needs to be investigated. 

Carbohydrate utilisation during prolonged endurance exercise is influenced by numerous factors, including the intensity and duration of the exercise, environmental conditions and the availability of endogenous and exogenous CHO (for reviews, see [34,35]). While muscle glycogen stores can be supercompensated by diet and training protocols to increase their contribution to muscle substrate use [36], the rate of oxidation of exogenous CHO from sources consumed prior to and during exercise is affected by the type and amount of CHO intake, and by prior adaptation of the gut via repeated exposure to enhance tolerance and intestinal absorption [37,38,39]. Although evidence for this ‘training’ effect has only been investigated using glucose (demonstrating upregulation of its transport protein, SGLT1), fructose absorption via GLUT5 is also known to upregulate rapidly following CHO exposure [39]. Additionally, the ingestion of artificial sweeteners such as sucralose can also increase SGLT1 content in animal models [40] via a cascade initiated by sweet taste receptors in the mouth and small intestine. Together, these strategies may enhance CHO availability and oxidation during prolonged high-intensity endurance exercise, leading to an improvement in exercise economy. 

Accordingly, the aim of this project was to undertake separate investigations of two different dietary approaches to enhance exercise economy in elite athletes during prolonged exercise simulating race pace in endurance (race walking) events. The first strategy (Beetroot Juice; BRJ) involved nitrate supplementation, using a newly modified protocol suited to endurance sports: a 2 d pre-load combined with an acute BRJ protocol providing a total dose (~19 mmol) known to achieve a physiological effect [41], but consumed before and during exercise [22]. The second strategy (Carb Max) was a multi-pronged protocol involving chronic diet-training adaptations and acute race strategies to maximise oxidation of endogenous and exogenous CHO sources during exercise. Our comparison (control) condition involved current sports nutrition guidelines at the “low end” of recommended CHO intake ranges [42], since this often represents real life practice [43,44]. Although we recognised that the multiple treatments in our second strategy would prevent us from identifying the specific contribution of any single element, we considered this to be a proof-of-concept study in which the likelihood of detecting changes to substrate use and exercise economy were highest. If such an outcome was achieved, it would then merit separate investigation of the individual components.

## 2. Materials and Methods 

### 2.1. Overview and Participants 

Twenty-one elite male race walkers participated in one of two training camps (January or May) held at the Australian Institute of Sport (AIS). The cohort, which included 16 athletes of high-level international representation (e.g., Olympic Games, World Championships, World Walking Cup) and five national level training partners, were recruited via targeted invitations from key athletes and the coach with whom the study was planned. This study conformed to the standards set by the *Declaration of Helsinki* and was approved by the Human Research Ethics Committee of the AIS (no 20171203). All subjects provided informed consent after being provided with study details both verbally and in writing. 

**Figure 1:**
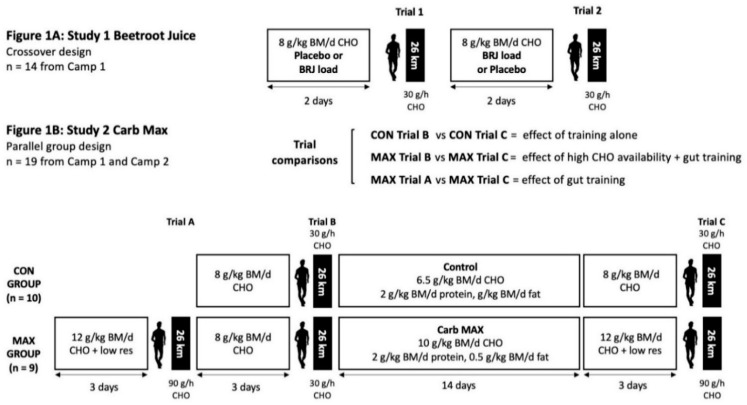
Schematic of the two investigations (Beetroot Juice: Figure 1A and Carb Max: Figure 1B) scheduled within a 4 week training camp involving 21 elite male race walkers. BM: body mass; BRJ: beetroot juice; CHO: carbohydrate; CON: control condition; low res: low residue/fibre diet; MAX: Carb Max intervention.

During the training camps, held over a ~4 week duration, athletes were accommodated at the residential facilities at the AIS to allow all training to be supervised and to permit strict dietary control for the various study interventions [45]. Participants had been undertaking base phase endurance training prior to the training camp, and the weekly training program (typically, 90–140 km/week) represented an intensified training block prior to early season competition. It consisted of a number of compulsory group race walking sessions, some sessions undertaken as intervention trials and the opportunity for athletes to complete additional sessions of race walking, gym and cross-training. The 4 week schedule was arranged to accommodate the investigation of two separate dietary interventions for exercise economy: Study 1: Beetroot Juice study and Study 2: Carb Max (see Figure 1 for summary of protocol). Overall, 15 athletes attended the first camp, while an additional 6 athletes completed the second. Figure 1 summarises the design of each study and the number of participants who completed each investigation. Characteristics of athletes who contributed to each part of the project are summarised in Table 1.

### 2.2. Overview of Dietary Control and Interventions 

Dietary treatments provided in this project were implemented using methods previously described elsewhere in detail [45]. Briefly, all foods and fluids consumed during the study were prescribed and provided by a team of chefs, food service dietitian and sports dietitians. Menus were constructed for the various phases of each study, and then meal plans were individually developed for each athlete to integrate personal food preferences and nutrient targets within his allocated dietary treatment. Energy intake was set to allow a small energy deficit and body mass (BM) loss of ~1 kg over the 4 week camp, as is normal for this stage of the athlete’s season. Baseline daily energy intake was set at 225 kJ/kg BM but adjusted according to individual feedback regarding training load and hunger. 

Chef-prepared meals were provided to athletes in a group dining setting, with individualised meals being served to each athlete and their intake recorded using calibrated scales accurate to 2 g. Individualised snacks were provided for intake between meals and before/during training sessions, with the requirement for their consumption to be cross-checked at the next meal. A range of “free foods and drinks” (foods with low energy such as fruits and vegetables, tea/coffee, water and a limited quantity of artificially sweetened beverages) were provided in the participants’ living area with a checklist to allow each participant to report on his day’s intake at the first meal of the following day. Nutrition support during longer training sessions and after key sessions was provided at the training site by members of the research team and intake was recorded. 

### 2.3. Physiological Testing before and after the Studies 

At the beginning (Baseline Testing) and the end (Post Testing) of each camp, all athletes undertook assessments of body composition via dual X-ray absorptiometry (DXA), resting metabolic rate (RMR) and aerobic capacity ($\dot{V}$O_2peak_) to investigate changes over the duration of the 4 weeks. The DXA was conducted using a standardised protocol previously described [46] on an iDXA (GE Healthcare, Milwaukee, WI, USA) with image analysis (enCore v16, GE Healthcare). RMR was assessed according to standardised outpatient protocols described elsewhere [47]. The incremental exercise test was identical to protocols previously reported [26]. Briefly, this involved four submaximal walking stages, each lasting 4 min and increasing in speed by 1 km/h each stage. After a 5 min rest, athletes then commenced an incremental ramp protocol, where speed increased every 30 s by 0.5 km/h for 4 min, after which time speed was fixed and gradient increased by 0.5% every 30 s until exhaustion. Respiratory gases were collected and analysed on a custom-built indirect calorimetry system (AIS, Canberra [48]). These tests were repeated prior to the final experimental trial (post testing).

### 2.4. Study One (Beetroot Juice)

#### 2.4.1. Overview

Fourteen race walkers (Table 1) completed this study during the January camp. In a crossover design, each athlete completed two 26 km race walking exercise protocols after a two-day nitrate or placebo loading protocol (see Figure 1A). In the 48 h prior to each experimental trial, athletes followed a standardised diet providing a daily intake of 225 kJ/kg BM, 8 g/kg CHO, 1.8 g/kg protein and 1.5 g/kg fat (see Figure 1A). Menus were constructed around CHO-rich foods that were naturally low in ^13^C, due to the use of a ^13^C tracer within Study Two. These diets were inclusive of the consumption on each of the two evenings before the experimental trial (−36, and −12 h) of either a 70 mL shot of NO_3_^−^-rich beetroot juice (BRJ), containing 6.45 mmol NO_3_^−^ (Beet-It Sport Shot, James White Drinks, Ipswich, UK) or an identical placebo supplement (PLA) in which the NO_3_^−^ content had been selectively removed. 

On the morning of the experimental trial, athletes arrived at the laboratory and an indwelling cannula was placed into a forearm vein to collect blood samples for subsequent determination of nitrate and nitrite concentrations. A resting sample was taken. Participants were then provided with a standardised breakfast (2 g/kg CHO), which included either 140 mL (~12.9 mmol NO_3_^−^) of BRJ supplement, or in the PLA condition, an empty capsule. The use of the capsule as a PLA treatment before/during the exercise protocol was necessary because the PLA condition doubled up as a preparation trial within Study 2 to test background ^13^C within exogenous CHO oxidation. Although this meant that the nitrate supplementation trials were not fully blinded, participants were told that the capsule provided an alternative form of NO_3_^−^. Furthermore, we note that the focus of the investigation was exercise economy rather than performance, an outcome that was unlikely to be influenced by belief effects. 

Two hours after breakfast, a second venous blood sample was collected, then athletes consumed 210 mL of an 8% CHO drink (2:1 glucose:fructose blend) before commencing the 26 km race walking protocol. After 7 km, at the end of the second treadmill component, a second treatment of either 70 mL BRJ (6.45 mmol NO_3_^−^) supplement or a PLA capsule was consumed, providing a total nitrate dose of ~19 mmol around the exercise session. Here and at the conclusion of each treadmill component, subjects were scheduled to consume 190 mL of the 8% CHO drink to provide an hourly CHO intake of ~30 g. This occurred as planned in the PLA trial, but in the BRJ trial, the CHO content and fluid volume of drinks at the 7 km and 13 km station were adjusted to take into account what had already been provided by the BRJ shot. Venous blood samples were collected at 7 km, 19 km and immediately following exercise to monitor nitrate and nitrite concentrations. 

#### 2.4.2. 26-km Exercise Protocol

The exercise protocol was conducted as a hybrid laboratory field test, with 0–1, 6–7, 12–13, 18–19 and 24–26 km being completed inside the laboratory on a motorised treadmill. The remaining distance was undertaken on an outdoor course (~5 km loops) at a consistent speed nominated by the athlete and maintained for each of their trials. Transition from the field to the laboratory was typically <30 s, with participants being able to race walk right to the door of the laboratory and proceed directly to a treadmill. Throughout the outdoor segment of the protocols, two aid stations were made available at ~2 km intervals to allow athletes to consume water *ad libitum*. Heart rate (HR) was monitored continuously throughout the trial (Polar Electro, Kempele, Finland).

The indoor exercise segments allowed standardised collection of samples and exercise metrics, while also providing an opportunity for controlled intake of test beverages. The treadmill component was completed at either 12 or 13 km/h, which equated to ~75% of the athletes $\dot{V}$O_2peak_ and approximated their 50 km race pace. However, to replicate the top-end finishing speed that typically occurs towards the end of a race walking event [49], speed during the final (26th) km was increased by 1.3 km/h (to 13.3 or 14.3 km/h). During the final 1 min of each kilometre, respiratory gases were collected into two Douglas bags (30 s each). Respiratory gases were analysed using O_2_ and CO_2_ analysers (AMETEK, Inc., Pittsburgh, PA, USA) to determine the gas fractions used to calculate oxygen consumption. Rates of total CHO and fat oxidation (g/min) were calculated from $\dot{V}$CO_2_ and $\dot{V}$O_2_ values using non-protein RER values [50]. These equations are based on the premise that $\dot{V}$O_2_ and $\dot{V}$CO_2_ accurately reflect tissue O_2_ consumption and CO_2_ production, and that indirect calorimetry is a valid method for quantifying rates of substrate oxidation in well-trained athletes during strenuous exercise of up to 85% of $\dot{V}$O_2peak_ [51]. At the end of each treadmill km, a fingertip blood sample was collected and analysed for blood lactate concentrations (Lactate Pro 2, Akray, Japan), while Ratings of Perceived Exertion (RPE, 6–20, Borg Scale) were collected. Prior to leaving the laboratory after each treadmill portion of the 26-km protocol, athletes consumed 190 mL of their test drink under supervision.

#### 2.4.3. Venous Blood Analysis—NO_2_^−^ and NO_3_^−^

Five venous blood samples per trial were collected into 3 mL lithium-heparin tubes. Samples were immediately centrifuged at 1500× *g* at 4 °C for 10 min, aliquoted and frozen at −80 °C until batch analysis was performed for NO_2_^−^ and NO_3_^−^. Plasma NO_2_^−^ was quantified by gas phase chemiluminescence (Sievers nitric oxide analyser 280i, Analytix Ltd., Durham, UK). Plasma was deproteinised 1:1 with ice-cold acetonitrile before injection into a glass purge vessel containing an acetic acid and sodium iodide solution, which reduces NO_2_^−^ to nitric oxide (NO) gas. Quantification of NO was determined by the detection of light emitted during the production of nitrogen dioxide formed upon reaction of NO with ozone, with luminescence detected by a thermoelectrically cooled, red-sensitive photomultiplier tube. The concentration of NO_2_^−^ was determined by plotting signal area (mV) against serial dilutions of sodium NO_2_^−^. Plasma NO_3_^−^ was deproteinised by centrifugation through a 30 kDa molecular weight filter (Pall Nanosep, Pall Corporation, Show Low, AZ, USA), and analysed spectrophotometrically. 20 µL of sample was added to a 96 well plate, followed by 80 µL ddH_2_O, 100 µL 0.1 M vanadium (III) chloride in 1 M hydrochloric acid, and 100 µL Greiss reagent (sulphanilamide, 2% *w*/*v* in 5% HCl, and N-(1-naphthyl)ethylenediamine dichloride, 0.1% *w*/*v* in water, mixed in equal quantities immediately before use). Following a 30 min incubation at 37 °C, absorbance was read at 540 nm on a plate reader (Enspire, Perkin Elmer, Waltham, MA, USA). The concentration of NO_3_^−^ was determined by plotting absorbance against serial dilutions of sodium NO_3_^−^.

### 2.5. Study Two (Carb Max)

#### 2.5.1. Overview 

Nineteen highly trained male race walkers participated in Study Two. Thirteen athletes participated in the January camp and an additional six athletes from the second training camp in May. In a parallel groups design, athletes were allocated to either a moderate CHO diet (control: CON; *n* = 10) or a novel, multi-pronged experimental approach to increase CHO availability and utilisation during exercise (Carb Max: MAX; *n* = 9). Dietary allocations were made with consideration to the athlete’s preference, while also matching for key characteristics including age, $\dot{V}$O_2peak_ and personal best race times (Table 1). Substrate use and exercise economy in the 26 km race walking protocol were assessed before and after the implementation of these treatments (Figure 1B: Trials B and C). Athletes assigned to the MAX intervention completed an initial 26 km race walking trial, where acute strategies relating to high CHO availability and utilisation were implemented for three days and examined prior to the implementation of the chronic diet-training adaptation component of the intervention (Figure 1B: Trial A). Thereafter, participants completed the pre-intervention trial, in which exercise characteristics were examined after three days of standardisation to the control treatment in both groups (Trial B). Athletes then commenced a 2 week training phase, in which the multi-pronged strategy to chronically increase CHO availability and utilisation was implemented for the MAX group. During this time, athletes followed a supervised, semi-structured training program, inclusive of weekly compulsory sessions of two long walks (>25 km), an interval training session (10 × 1000-m efforts repeated on a 6-min cycle) and a tempo hill session (14 km, ~450 m elevation). All other training was completed at the athlete’s discretion and recorded into an electronic training diary. After the 14 d intervention, the post-testing period was commenced with the MAX group receiving acute strategies to increase endogenous and exogenous CHO availability for the final exercise protocol (Trial C). 

#### 2.5.2. Summary of Dietary Intervention for Study Two

Dietary conditions for the CON trials (Trials B and C), and MAX trial B consisted of 72 h of a standardised glycogen storing “race preparation” diet; this provided ~225 kJ/kg BM, 8 g/kg CHO, 1.8 g/kg protein and 1.5 g/kg fat (see Figure 1B). On the morning of each trial, a pre-exercise meal providing 2 g/kg CHO was consumed 2 h prior to the start of exercise. Just prior to the start of exercise, athletes consumed 210 mL of an 8% CHO drink (2:1 glucose:fructose blend). Throughout the exercise protocol in the CON trial, athletes consumed 190 mL of this drink at 6 km intervals to provide ~30 g/h CHO (see Figure 1B). During the 2 week intervention period, athletes in the CON group were provided with a diet providing ~225 kJ/kg BM, 6.5 g/kg CHO, 2 g/kg protein and 2 g/kg fat. Intake of CHO during longer training sessions was maintained at ~30 g/h. These strategies represent the low end of the range of guidelines for CHO intake for endurance athletes for training, and fuelling targets before and during a long distance race [42]. 

The MAX dietary intervention combined a chronic training strategy (to adapt the muscle and gut to train with high CHO availability) with an acute dietary protocol implemented prior to and during the exercise protocol (to acutely maximise endogenous and exogenous CHO availability). The 14 d chronic intervention consisted of an energy- and protein-matched diet with CHO content increased to meet the high end of the range of guidelines for CHO intake in the training diet [42]: ~225 kJ/kg BM, 10 g/kg CHO, 2 g/kg protein and 0.5 g/kg fat (Figure 1B). Dietary strategies to increase CHO content included providing a substantial serve of potatoes as the key CHO source at a meal (e.g., 300 g potato wedges or 500 g mashed potato), increasing the serve sizes of grain products, juices and sweetened dairy products at meals and snacks, and increasing training-focused CHO choices (drinks, gels, confectionery). Strategies to adapt the gut to enhance CHO absorption included (1) increased CHO intake consumed during training sessions to 90 g/h, with specific use in week two of the 2:1 glucose:fructose drink used in the trial protocol and (2) implementation of 5 mg/kg BM/d of sucralose provided in a 2 mM solution and ingested three times per day between meals in an attempt to increase intestinal glucose uptake. 

As shown in Figure 1B, MAX athletes undertook two pre-intervention trials to compare the total effects of the MAX diet (acute and chronic elements—Trial C) with the control diet (Trial B), as well as an investigation of the acute intervention, with and without the 2 w adaptation to the high CHO availability diet (Trial A vs. Trial C). While CHO intake during the 72 h preparation (~8 g/kg/d) and in-trial protocol (30 g/h) for Trial B was the same as the CON group, MAX Trials A and C consisted of an aggressive CHO loading protocol in which the CHO content was further raised to a target of ~12 g/kg BM, with a slight decrease in fat and protein content to allow energy intake to remain similar. The fibre content and volume of the diet was reduced by the removal of wholegrain cereals, and replacement of fresh/uncooked fruit and vegetables with juices, fruit purees or well-cooked vegetables without skins (e.g., potatoes). On the morning of Trials A and C, the standardised pre-exercise meal providing 2 g/kg CHO was consumed 2 h prior to the start of exercise. Just prior to the start of exercise, athletes consumed 210 mL of a 24% CHO drink (2:1 glucose:fructose blend). Throughout the exercise protocols, athletes consumed 190 mL of this drink at 6 km intervals to provide ~90 g/h CHO (see Figure 1B). 

#### 2.5.3. 26-km Exercise Protocol

Each experimental trial followed the same exercise protocol undertaken in Study One (see above). However, in Study Two, different amounts of CHO were consumed across trials (30 g/h vs. 90 g/h), and additional study techniques were undertaken to investigate oxidation of endogenous and exogenous CHO during exercise (see below). To facilitate this, the trial drinks were labelled with ^13^C glucose and fructose, and an additional sample of venous blood and expired air were collected during each of the km stages undertaken on the treadmill for determination of serum glucose concentrations and ^13^C breath analysis, respectively. Specifically, CHO beverages consumed during the trials were enriched with 0.2 mg/g of ^13^C uniformly labelled glucose and 0.1 mg/g ^13^C uniformly labelled fructose (d-glucose and d-fructose, U-^13^C6, 99%, Cambridge Isotope Laboratories, Tewksbury, MA, USA). The final enrichment of the 8% and 24% CHO beverages were 297.7 ± 18.5‰ vs. Pee Dee Bellemnitella (PDB) and 93.7 ± 6.2‰ vs. (PDB), respectively.

The seven venous blood samples were collected into 2 mL serum separator tubes during each trial. Samples were left to clot on the bench top for 30 min prior to centrifugation at 1500× *g* at 4 °C for 10 min. Serum was then immediately analysed for blood glucose concentrations with a COBAS Integra 400 automated biochemistry analyser (Roche Diagnostics, Rotkreuz, Switzerland).

#### 2.5.4. ^13^C Breath Analysis

Once expired air samples had been collected into Douglas Bags during the last minute of each treadmill section, duplicate 10 mL air samples were collected in uncoated evacuated tubes (Grenier Bio One, Kremsmünster, Austria). These samples were analysed for ^13^C:^12^C ratio by gas chromatography (GC) isotope ratio mass spectrometry (IRMS). Briefly, gas samples were passed through a packed GC column at 60 °C with the resultant chromatographic peak passed into an IRMS (Europa Scientific Hydra 20-20, Sercon Ltd., Crewe, UK) where the isotopomers at m/z 44, 45, and 46 for CO_2_, were measured. The δ^13^C value was determined in relation to the reference gas; IA-CO2-7 (δ^13^C = −38.48‰ vs. V-PDB).

Exogenous CHO oxidation (CHO_Exo_, in g/min) was calculated using the formula [52]:

$${\mathrm{CHO}}_{\mathrm{Exo}}=\;\dot{V}{\mathrm{CO}}_{2}\;[(\delta \exp-\delta \exp_{\mathrm{bg}})/(\delta \mathrm{ing}-\delta \exp_{\mathrm{bg}})]/k$$

where $\dot{V}$CO_2_ is the rate of CO_2_ production (in g/min), δexp is the ^13^C enrichment of the expired air (in ‰), δexp_bg_ is the ^13^C enrichment of the expired air in the time matched baseline trial (in ‰), δing is the ^13^C enrichment of the ingested CHO drink (in ‰) and k is 0.747 L/g (i.e., the volume of CO_2_ produced, by the complete oxidation of one gram of glucose, at standard temperature and pressure). No correction was made for background ^13^C since the pilot protocol found this to be negligible.

Calculations were made assuming that during exercise at the intensities used in this study there is negligible loss of CO_2_ to the bicarbonate pool [53] once steady state is reached after ~60 min [54]. Therefore, the calculations of exogenous CHO oxidation may be underestimated at 1 km and 7 km. These data points are presented for completeness but have not been included in the statistical analyses undertaken in the study.

### 2.6. Statistical Analysis

All data are presented as mean (standard deviation). General linear mixed models were performed for each variable using the package “lme4” and the restricted maximal likelihood approach. Models were created to (1) compare BRJ and PLA supplementation conditions (Study One), (2) assess the impact of the CHO Max strategy compared to a more moderate CHO intake and (3) assess the acute compared to chronic implementation of the CHO Max strategy (i.e., gut training). Residual plots were used to assess homoscedasticity and normality, and where appropriate, data were log transformed. Where relevant, main effects were included for dietary intervention, trial and time point. Initial models included all possible interactions, however non-significant interactions were dropped from the final model for ease of interpretation. A random intercept for athlete and camp were included to adjust for baseline values and differences between camp. Furthermore, in Study Two, temperature and humidity were included within the model to account for different environmental conditions between trials. In Study Two, values for exogenous and endogenous CHO oxidation for the first hour of the exercise protocol were not included in statistical analyses due to the likely methodological under-estimation of true values. *p* values were obtained using type II Wald *F* tests with Kenward-Roger degrees of freedom as implemented in the R package car. Post hoc comparisons with Tukey adjustments were made using the R package “emmeans”. Significance was set at *p* < 0.05.

## 3. Results

### 3.1. Physiological Changes over the Training Camp

Physiological characteristics from Baseline and Post Testing of the 19 subjects who completed the full 4 weeks of the training camp are summarised in Table 2, showing the effects of the intensified training program and differences that might have occurred due to allocation to different dietary treatments in Study Two. Among this group, the mean weekly training volume was 138 km and 13.5 h (including gym and cross-training. Increases in $\dot{V}$O_2peak_ over the 4 weeks were evident (*p* = 0.018) and were similar between diet groups (*p* = 0.795). A decrease in BM occurred (~1.0 kg; *p* < 0.001), however there were no differences between dietary groups (*p* = 0.817). The change in fat mass in the MAX group was significant (−1.4 kg; *p* < 0.001) but failed to reach significance in the CON group (−0.7 kg; *p* = 0.068). No differences in fat free mass (FFM) were evident over time (*p* = 0.826) or between diets (*p* = 0.991). A decrease in absolute RMR (*p* = 0.034) and RMR expressed relative to FFM (*p* = 0.025) occurred during Post Testing, with no differences between diets (*p* > 0.05).

### 3.2. Results of Study One (Beetroot Juice)

**Figure 2:**
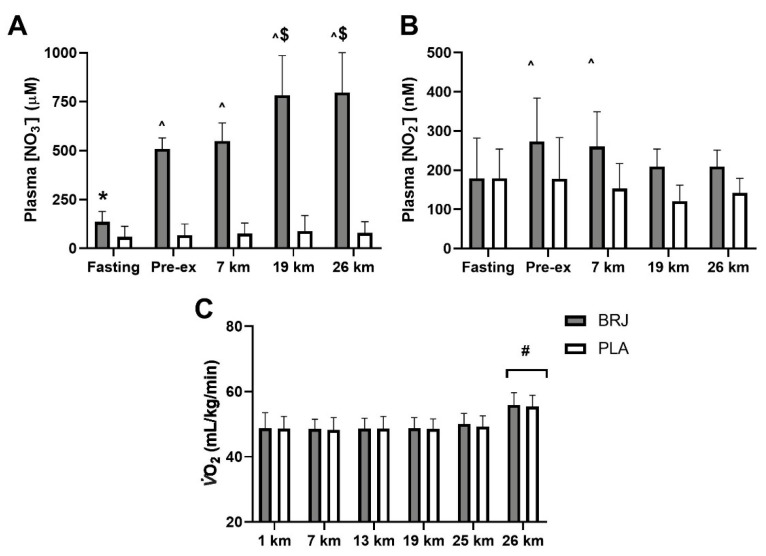
Plasma nitrate (NO_3_^−^) concentrations (**A**), nitrite (NO_2_^−^) concentrations (**B**) and Oxygen uptake (**C**) during the 26 km race walking protocol in athletes following a beetroot juice (BRJ) or placebo (PLA) intervention. Significance set at *p* < 0.05; * Different to PLA. ^ Increased above Fasting in BRJ only. ^$^ Increased from Pre-ex and 7 km in BRJ only. ^#^ Higher than all other time points.

Each group met dietary intake targets for the 48 h prior to each trial (data not shown). Results from the two trials involving the intake of BRJ or PLA before and during the 26 km race walking protocol are summarised in Figure 2 and Table 3. The intake of BRJ before and during the exercise protocol was well-tolerated. The 2-d pre-load of BRJ was associated with higher fasting concentrations of plasma NO_3_^−^ than in the PLA trial (Figure 2A, *p* < 0.001). Further intake of BRJ in the pre-exercise meal (−2 h) and during exercise at the 7-km mark resulted in a continued increase in plasma NO_3_^−^ concentrations relative to fasting, which was maintained for the rest of the trial (all *p* < 0.001). Plasma NO_2_^−^ concentrations (Figure 2B) were significantly increased at pre-exercise and 7 km compared to fasting levels in the BRJ condition only (*p* = 0.013). No changes in plasma [NO_3_^−^] (Figure 2A) or [NO_2_^−^] (Figure 2B) were observed in the PLA trial before or during exercise.

Rates of substrate utilisation across exercise (Table 3), showed a gradual reduction in CHO oxidation over time, followed by a significant increase at the 26 km time point as a result of the increase in speed/exercise intensity. There was a reciprocal change in rates of fat oxidation to match this. Heart rate and rates of perceived exertion (RPE) increased across time during the exercise session with a further significant increase at 26 km. Blood lactate concentrations increased at the onset of exercise and gradually declined to pre-exercise levels until the increase associated with higher intensity exercise at 26 km. Oxygen uptake during exercise (Figure 2C) remained stable across time, until the increase at 26 km associated with the increased speed (*p* < 0.001). No differences in oxygen uptake (*p* = 0.090), rates of CHO (*p* = 0.203) or fat (*p* = 0.818) oxidation, respiratory exchange ratio (RER) (*p* = 0.795), lactate concentrations (*p* = 0.052), heart rate (*p* = 0.095) or RPE (*p* = 0.633) were evident between the BRJ and PLA conditions (Figure 2C and Table 3). 

### 3.3. Results of Study Two (Carb Max)

Table 4 summarises the assessment of actual dietary intakes in Study Two, with a summary of mean daily intakes of the various dietary treatments in the two groups. The MAX dietary treatment was successfully implemented, and all athletes complied with their assigned dietary treatment and met the targeted energy and macronutrient intakes of each phase. There were no differences in energy and macronutrient intakes between trials or groups for the 72 h pre-trial standardised protocols involving moderate CHO availability (Trials B and C for the CON group and Trial B for the MAX group). During the 14 d intervention phase, as directed, CHO intake was higher in the MAX group than the CON group (*p* < 0.05), while fat intakes were lower, and protein and fibre intakes were similar. Energy intake (kJ/kg/d) was slightly but significantly higher in the MAX group than CON during this phase (*p* < 0.05). During the 72-h standardised protocol before the high CHO availability trials (MAX Trials A and C) there was an increase in CHO content (*p*< 0.05), and a reduction in fat, protein and fibre intake (all *p* < 0.05) compared with the moderate CHO availability trials. No differences in dietary intake were noted between the two high CHO availability protocols.

**Figure 3:**
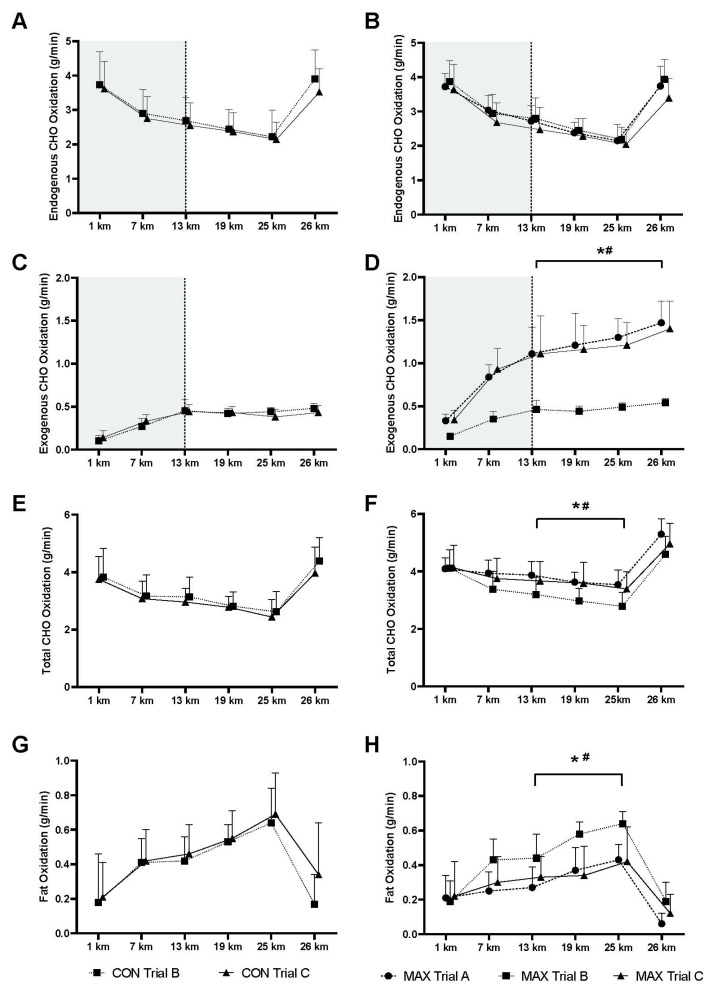
Calculated rates of oxidation of endogenous CHO (**A**,**B**), exogenous CHO (**C**,**D**) total CHO (**E**,**F**) and total fat (**G**,**H**) during a 26 km race walking protocol for elite male race walkers following moderate CHO availability (CON; *n* = 10; **A**,**C**,**E**,**G**) or high CHO availability (MAX; *n* = 9; **B**,**D**,**F**,**H**) intervention. * Significant within group difference between Trial A and B. ^#^ Significant within group difference between Trials B and C. Note that attention has been limited to interaction effects, and in the case of exogenous CHO (and endogenous CHO, calculated by difference from total CHO), only values from 13–26 km have been included in the statistical analysis.

Changes in substrate utilisation during the exercise protocols are summarised in Figure 3 with calculated rates of endogenous (Figure 3A,B), exogenous (Figure 3C,D) and total CHO oxidation (Figure 3E,F) and fat oxidation (Figure 3G,H). Main effects of time and exercise intensity were evident, with a gradual decrease in total CHO oxidation and reciprocal increase in fat oxidation over the first 25 km of the session (*p* < 0.001), followed by a reversal of these changes with the higher workload at 26 km (*p* < 0.001). Rates of endogenous CHO oxidation (Figure 3A,B), identified by the difference between total and exogenous CHO values over the second hour of exercise for which the data are credible, decreased from 13–25 km, followed by a sharp increase at 26 km due to the increase in race walking speed (*p* < 0.001). However, there were no differences between trials (*p* = 0.272) or diets (*p* = 0.985). In the case of exogenous CHO (Figure 3C,D), differences in the patterns and overall rates of oxidation between groups and trials were only associated with the amounts of CHO consumed during the exercise protocol (i.e., MAX Trial A and C vs. MAX Trial B, Figure 3D). Specifically, there were no between-group differences in exogenous CHO oxidation during the moderate CHO availability trials (CON Trial B vs. MAX Trial B, *p* = 0.964) and no within-group differences between matching trials performed prior to and after the 2 week training and diet intervention (CON Trial B vs. C, *p* = 0.654; MAX Trial A vs. C, *p* = 0.995).

Within the CON group, rates of exogenous CHO oxidation (Figure 3C) were 0.44 ± 0.10 g/min at the beginning of the second hour of exercise (13 km) during which values are generally recognised as valid. There was insignificant (*p* = 0.992) increase to 0.46 ± 0.07 g/min during the final (26th) kilometre at the higher speed. A decrease in endogenous CHO oxidation (Figure 3A) over 13–25 km contributed to a net reduction in rates of total CHO oxidation over the session (*p* < 0.001, Figure 3E) and the increase in total CHO oxidation with the increase in speed at 26 km was attributable to an increase in endogenous CHO use. However, there were no differences in the rate of total CHO oxidation (*p* = 0.781) or fat oxidation (*p* = 0.909) between Trials B and C.

In the MAX dietary condition, there was no difference in rates of endogenous CHO use between trials (Figure 3B); however, the lower CHO intake during exercise in Trial B was associated with lower rates of exogenous CHO utilisation (Figure 3D compared to Trial A (*p* < 0.001) and Trial C, *p* < 0.001) from 13 km onwards. Maximal rates of exogenous CHO utilisation were achieved at the 26 km mark, 1.47 ± 0.25 g/min and 1.40 ± 0.32 g/min for Trial A and Trial C, respectively. The total rate of CHO oxidation (Figure 3F) was therefore significantly higher from the 13 km mark onwards in Trials A and C compared to Trial B (*p* = 0.026). In contrast, there was a greater increase in fat oxidation rates (Figure 3H) during the second half of Trial B compared to Trials A and C (*p* = 0.019).

Table 5 summarises oxygen utilisation, respiratory exchange ratio (RER), heart rate, RPE, and blood metabolites from the 26 km exercise sessions in Study Two. Relative $\dot{V}$O_2_ (mL/kg/min) remained similar across the steady state component of the 26 km race walk protocol (1–25 km), with a ~15% increase in $\dot{V}$O_2_ evident during the final kilometre at the higher speed (*p* < 0.001). There were no differences in $\dot{V}$O_2_ between trials (*p* = 0.915) or diets (*p* = 0.442). Blood glucose concentrations increased at the 7 km mark and remained elevated throughout exercise (*p* = 0.001); however, no differences between trials (*p* = 0.416) or diets (*p* = 0.913) were observed. RPE, blood lactate concentrations and HR gradually increased throughout the exercise sessions (all *p* < 0.001). HR was generally lower during Trial C compared to Trial B (*p* < 0.001). No differences between dietary conditions (MAX vs. CON) were evident for any variable (*p* > 0.05). 

## 4. Discussion

This project investigated two dietary strategies designed to reduce the oxygen cost of a race walking protocol which simulated event characteristics in elite male race walkers. The main outcomes of the investigation were: 1. A BRJ supplementation protocol designed for endurance exercise (2 day preload followed by intake 2 h before and during the first hour of exercise) achieved a sustained increase in blood nitrate concentrations throughout a ~2 h exercise session. However, this protocol failed to maintain the early elevation in plasma nitrite concentrations for the second half of the exercise and was not associated with a detectable change in the oxygen cost of exercise at speeds relevant to real-life races in these elite athletes; 2. A multi-pronged strategy to increase the availability of endogenous and exogenous CHO sources was associated with an increase in the total rate of CHO oxidation during the second half of a prolonged exercise protocol, principally via an increase in rates of exogenous CHO use. However, this was not associated with a detectable change in the oxygen cost of exercise; 3. Chronic “gut training” strategies purported to increase gut absorption and muscle oxidation of exogenous CHO were not associated with an increase in rates of exogenous CHO oxidation in these elite athletes.

Beetroot juice products have become a popular sports supplement due to their classification as an evidence-based performance aid [55]. Dietary nitrate has been shown to generate NO via an oxygen-independent pathway that is complementary to arginine-NO production and reliant on the oral microbiome [56]. Elevations in plasma nitrate peak about an hour after nitrate intake with plasma nitrite reaching peak elevation a further 1–2 h later [41], demonstrating both a dose-response relationship and the sequencing of events of gastrointestinal absorption of nitrate, sequestering by the salivary glands, reduction of nitrate in the saliva via reductases principally provided by oral bacteria, and subsequent swallowing and absorption of the nitrite into the circulation [56]. Many studies have followed up the early observations of a reduction in the oxygen cost of submaximal cycling with nitrate supplementation [16,17]. Indeed, a meta-analysis of such studies conducted up until 2015 reported an overall effect size of 0.26 (0.15–0.38, *p* < 0.0001) for the reduction in oxygen utilisation during sub-maximal exercise, with effects being greatest during moderate and heavy intensity exercise [57]. Variability in findings around effects of nitrate supplementation on both economy and performance can be explained, in theory [19,56] and by meta-analysis, by a number of factors including the supplementation protocol (amount, type and timing) [58], the type of exercise [57] and the training status and aerobic fitness of the subject group [59,60]. The apparent lack of effect in elite endurance athletes may be a function of their athletic status (e.g., due to a superior background NO production or reduced number of Type II muscle fibres in which nitrate supplementation might be most suited to targeting conditions of hypoxia or acidosis [19,56,61,62]) as well as an interaction with the conditions of use. Nevertheless, it is possible that the optimal protocol in concert with the right scenario of use might still include some benefits for elite athletes [19,56,63]. 

The current study attempted to integrate some of the factors likely to promote the benefits of BRJ supplementation in elite endurance athletes. We employed a supplementation protocol that included a pre-load to increase baseline blood nitrate concentrations and, potentially, the muscle nitrate reservoir [64], ensured the absence of antibacterial agents that might interfere with the contribution of the oral microbiome to the entero-salivary pathway of nitrite generation [65] and used a pre-exercise dose previously shown to optimise plasma nitrate and nitrite concentrations and the resultant physiological responses [37]. Importantly, we also provided a “top up” dose of BRJ at ~35 min *during* exercise in an attempt to maintain the elevations in plasma nitrate and nitrate throughout the exercise task [22]. Our supplementation protocol was successful in increasing fasting nitrate concentrations above the placebo trial and in maintaining a sustained elevation in blood nitrate concentrations throughout the 26 km race walking protocol. However, unlike the study of Tan et al. [22], which employed a similar protocol for a ~130 min cycling task in recreationally active men (2 × 70 mL of the same nitrate-rich BRJ @ 2.5 h pre and 1 × 70 mL after 1 h of cycling), we did not observed a sustained elevation in blood nitrite concentrations for the duration of our exercise protocol. Specifically, although nitrite values were numerically higher than fasting concentrations in the BRJ trial, this only reached statistical significance for the first half of the 26 km race walking session. Of course, blood nitrite concentrations represent a balance between rates of generation from nitrate and rates of utilisation (e.g., NO production, reversal to nitrate) or uptake into tissues, so it is unclear whether the physiological effects of the BRJ supplementation were sustained throughout exercise. Furthermore, in contrast to the previous study [22], we did not see a drift in the oxygen cost of steady state exercise over the ~2 h in the placebo condition nor a reduction in the oxygen cost of exercise at any stage of our protocol. The lack of the drift in oxygen uptake over exercise and the failure to see any improvement in economy in association with nitrate supplementation may be related to the calibre and training status of the athletes and the relative intensity of the protocol. In any case, we conclude that in these elite athletes, BRJ supplementation did not provide a detectable change in exercise economy at speeds associated with real-life racing, which included a transition to a faster “finishing” or “critical moment” speed. Since we did not measure performance per se in this protocol, we are unable to speculate whether any changes would have occurred due to undetectable changes in economy or other mechanisms. 

The Carb Max protocol developed for this study combined a range of strategies to increase CHO availability from both exogenous and endogenous sources, via the interaction of chronic and acute effects. An aggressive glycogen supercompensation protocol was undertaken prior to the high CHO availability Trials A and C. Here, 72 h of very high dietary CHO (>11 g/kg BM/d) was achieved, and in the case of Trial C, was superimposed on a chronic (14 day) intake of CHO at the high end of the range of guidelines recommended for daily refuelling in endurance athletes [42]. Unique features of this well-tolerated dietary treatment included a focus on a high intake of potatoes as a CHO source, rather than the usual focus on grains or sugar-rich foods. Furthermore, a reduction in the fibre/residue content of the CHO-loading diet allowed the acute increase in CHO targets to be met without gastro-intestinal discomfort. Anecdotally, this strategy is integrated by endurance athletes into CHO loading protocols to offset the increase in BM due to the weight of additional muscle glycogen and water storage by reducing the weight of undigested food in the gut [4]. Although it is impractical to undertake biopsy-derived measurements of muscle glycogen in elite athletes to confirm whether the combined strategies elevated muscle glycogen stores above values achieved in the control diet or the pre-intervention phase of Carb Max treatment (Trial A), previous studies in well-trained athletes have identified higher muscle CHO storage when CHO intake was increased for several days from ~10 g/kg/d to 12 g/kg/d within the same energy content [66]. The chronic intervention associated with Carb Max, which increased total dietary CHO within daily energy intake, was likely associated with better restoration of daily muscle glycogen stores throughout the intensified training block [67]. Whether this supports better quality training, different physiological adaptations or different performance outcomes is beyond the scope of the current project and is a complex and nuanced issue [7]. Nevertheless, even if the acute CHO loading protocol alone achieved greater muscle glycogen storage prior to each of the MAX Trials A and C, this was not associated with a greater contribution of endogenous CHO stores to total CHO oxidation, at least during the second hour of the exercise protocol in which tracer methodology provides more credible data. In addition, there was no evidence of changes in the contribution of endogenous CHO stores to substrate utilisation during exercise as a result of the 2 week adaptation to higher dietary CHO intakes. 

Another characteristic of the MAX treatment was that it provided an acute increase in the availability of exogenous CHO during exercise in Trials A and C. A CHO feeding regimen providing 90 g/h of a glucose:fructose mixture during the 2 h race walking session was compared with the control protocol of 30 g/h CHO intake, with the latter representing the lower end of the range recommended in current sports nutrition guidelines for endurance events [42]. The rate of intake of CHO during exercise is a key factor in determining exogenous CHO oxidation, with the intestinal absorption of CHO being the limiting factor in gut delivery and subsequent utilisation in the muscle. The use of “multiple transportable CHOs” (combinations of CHO sources with non-competing intestinal absorption mechanisms) represents a solution to this gut bottleneck and has been associated with high rates of exogenous CHO oxidation during laboratory exercise (for review, see [68,69]) as well as successful performance in endurance events in real-world sports [70,71]. In the current study, increased CHO intake during exercise in the MAX trials was associated with higher rates of total CHO utilisation via the increased rate of exogenous CHO oxidation in the second hour of exercise. Indeed, peak rates of exogenous CHO utilisation during the race walking protocol were ~1.45 g/min compared with ~0.45 g/min in the control conditions. Despite these differences in CHO oxidation, there were no differences in the oxygen cost of exercise between trials or over the course of exercise, apart from the increased oxygen utilisation at 26 km associated with the higher speed. 

In addition to failing to find a difference in the oxygen cost of exercise associated with small but significant changes in substrate utilisation, we did not observe the expected effect of the chronic element of the Carb Max treatment on exogenous CHO use. Specifically, there was no detectable change in rates of exogenous CHO oxidation after the 2 week adaptation to strategies that are known, or purported, to increase intestinal CHO absorption. “Gut training” via repeated exposure to CHO consumption during exercise sessions has been shown to increase the intestinal absorption and oxidation of exogenous CHO as an exercise substrate (for review, see [39]). This has been attributed to an upregulated expression of the intestinal glucose transport protein, SGLT1, and potentially, a similar effect on the fructose transporter, GLUT5 [39]. Interestingly, although the functional outcomes of enhanced CHO utilisation have been observed following gut training for CHO in general [38,72], there seems to be an enhanced effect when applied to the specific type of CHO and exercise that have been practice [38]. Although such elements were integrated into our study design, we did not detect an increase in exogenous CHO oxidation after 2 weeks of targeted gut training. 

An additional strategy included in our study, albeit with tentative benefits which were unable to be distinguished independently within the study design, was the repeated intake of the non-caloric sweetener, sucralose, throughout the 2 week chronic adaptation period. It has been suggested, based on animal and clinical studies, that non-caloric sweeteners might activate G-protein coupled receptors T1R2 and T1R3 in the mouth and intestine, initiating the sweet taste signal transduction cascade and upregulating intestinal CHO transporters (for review, see [73]). Although a previous study providing acute exposure to sucralose (1 mM) just prior to exercise failed to find an effect on the oxidation of CHO subsequently consumed throughout the protocol, it was suggested that further studies investigate higher doses and repeated/chronic use [73]. Such treatments (2 mM sucralose, consumed 3 times a day for 2 weeks) were included in the current study, but in the absence of an overall change in rates of exogenous CHO oxidation as a result of our multi-pronged approach to “gut training”, we must conclude that this strategy was ineffective. 

Although our inability to detect a benefit of gut training on exogenous CHO utilisation in the current study may appear unexpected, a likely explanation is that our participants, elite race walkers, already practice regular intake of CHO during exercise as a training culture supported by the logistics of their competitive events in which feedzones are provided every 2 km [4]. It is possible that habitual practices had allowed each individual to optimise his gut function for this purpose. Indeed, the pre-intervention rates of exogenous CHO use in our population (mean peak of 1.47 g/min) are high in absolute comparison to findings from other studies using similar tracer technology (~1.2–1.4 g/min) [68,69]. Furthermore, when expressed as an oxidation index [74], such a rate represents 98% of the intake of 90 g/h, supporting the hypothesis that these athletes were already at the ceiling of gut absorption capacity.

We note that the primary outcome variable of this study, the economy of race walking, represents a proxy measure or contributor to performance, rather than being a performance measure per se. Furthermore, this metric is reliant on the measurement of steady state oxygen consumption using various types of manual (i.e., Douglas bag method) or automated (i.e., commercial breath-by-breath analysers) gas-analysis systems [75]. The methodology used in this study involved a bespoke system, constructed specially for the Australian Institute of Sport to suit the logistics of testing groups of athletes as well as to maximise the reliability and validity of measurements of pulmonary gas-exchange data. This involves a custom-designed and -built open-circuit indirect calorimetry system with associated in-house software, and in the specific case of this study, manual collection of respiratory gases via Douglas bags. The typical error of measurement associated with this system and its best practice protocols of use is ~2.4% [48], which is half of the values previously reported for criterion Douglas Bag methods or the most accurate commercial metabolic cart systems [76]. 

Our techniques offered us better precision in measuring changes in oxygen utilisation during exercise than most standard or available systems. However, we acknowledge that we may not have been able to detect minor changes in economy of exercise that could be important to the performance of elite sport, where daily variability of exercise metrics within athletes may be reduced and margins between winning and losing are incredibly small [77]. Indeed, although it is possible to detect real changes in economy of 2% with protocols similar to those used in this study [78], real effects smaller than this require repeated measurements on an individual and/or appropriately large sample sizes for detection [79]. According to real-life measurements and models of running economy, even minor changes of this order of magnitude could achieve changes in performance that would be considered worthwhile to substantial in elite distance running events [11,79]. Similar investigations in race walking which calculate the translation of changes in economy to changes in expected race speeds are currently lacking. Therefore, while our results suggest that neither BRJ supplementation nor increases in the contribution of CHO achieve a measurable change in oxygen utilisation during prolonged race walking which simulated important characteristics of competition, more subtle alterations may be beyond our detection capabilities. Furthermore, since both strategies can enhance sports performance via mechanisms other than changes to exercise economy, further investigation of their possible benefits in elite endurance sport is warranted. 

## 5. Conclusions

Given the important of exercise economy in the performance of endurance exercise events, we implemented two strategies purported to reduce the oxygen cost of exercise with rigorous dietary and exercise control in elite male race walkers. Within the limits of detection of changes of our study design and methodology, we failed to find any changes in oxygen use during prolonged exercise related to race speeds following a BRJ supplementation protocol tailored to meet the needs of endurance sport (2 d pre-load, pre-exercise intake and top-up during exercise). Similarly, a “Carb Max” intervention combining chronic and acute strategies to increase endogenous and exogenous CHO availability also failed to alter oxygen utilisation during the exercise protocol despite increasing the contribution of CHO oxidation, a more economical muscle fuel source, to muscle energy needs. The chronic diet, featuring a very high contribution of CHO to its energy content was well tolerated, even when a specific CHO-loading protocol to further increase CHO intake was superimposed prior to the exercise trial. However, increased CHO oxidation associated with the Carb Max treatment largely came from a greater contribution of exogenous CHO oxidation; this was achieved by increasing intake from multiple transportable CHO sources during exercise from 30 g/h to 90 g/h. Specific gut training activities integrated into the chronic Carb Max diet failed to achieve an increase in exogenous CHO oxidation via the expected enhancement of intestinal CHO absorption. However, since the baseline values of exogenous CHO use were already high (peak values of ~1.47 g/min and an oxidation index >90%), it is likely that the habitual practices of these elite athletes had already optimised gastrointestinal capacity for CHO delivery. Future studies should investigate the efficacy of these treatments in less elite/well-trained cohorts who may be below the ceiling for maximal capacity of systems that promote high exogenous or endogenous CHO availability. Furthermore, investigations of BRJ supplementation and strategies to enhance CHO oxidation in elite athletes should continue, since these interventions may enhance performance through mechanisms unrelated to exercise economy.

## Figures and Tables

**Table 1:** Characteristics of 21 elite male race walkers who participated in this project.

	Study One: BRJ	Study Two: Carb Max	
Characteristics	(*n* = 14)	CON (*n* = 10)	MAX (*n* = 9)
Age (y)	30.7 (4.2)	29.4 (4.6)	29.7 (4.2)
Body Mass (kg)	67.9 (4.7)	68.4 (9.4)	68.7 (5.0)
$\dot{V}$O_2peak_ (mL/kg/min)	63.9 (5.5)	60.9 (5.3)	63.1 (4.6)
10 km personal best min:sec.00)	40:45.32 (1:02.50)	41:11.21 (1:33.31)	40:55.00 (1:03.36)
20 km personal best (hr:min.sec)	1:22.53 (0:02.03)	1:24.29 (0:04.45)	1:23.04 (0:01.59)

Data are reported as mean (standard deviation).

**Table 2:** Change in physiological characteristics over ~4 week training camp in 19 elite male race walkers.

	Baseline Testing		Post Testing	
	CON (*n* = 10)	MAX (*n* = 9)	CON (*n* = 10)	MAX (*n* = 9)
$\dot{V}$O_2peak_ (mL/kg/min)	60.9 (5.3)	63.1 (4.6)	63.1 (4.1) *	64.1 (4.6) *
Body Mass (kg)	68.4 (9.4)	68.7 (5.0)	68.2 (9.9) *	67.3 (4.6) *
Fat Mass (kg)	8.7 (1.9)	9.3 (2.8)	7.8 (2.2)	7.9 (2.3) *
Fat Free Mass (kg)	60.3 (8.8)	60.0 (5.4)	61.0 (8.8)	60.1 (5.0)
RMR (kJ/d)	7992 (1017)	7597 (427)	7614 (1168) *	7391 (573) *
RMR (kcal/kg/FFM)	133 (13)	127 (9)	126 (9) *	123 (10) *

Data are reported as mean (standard deviation); * Significantly different to baseline: *p* < 0.05. $\dot{V}$O_2peak_: peak aerobic power; RMR: resting metabolic rate; FFM: fat free mass; CON: control condition; MAX; Carb Max condition.

**Table 3:** Physiological responses during a 26 km race walking protocol following intake of beetroot juice (BRJ) or nitrate-depleted placebo (PLA) in 14 elite male race walkers.

		Pre	1 km	7 km	13 km	19 km	25 km	26 km
Body Mass (kg)	BRJ	69.8 (6.4)						68.2 (6.3)
	PLA	68.7 (5.4)						67.6 (6.1)
CHO Ox (g/min)	BRJ		3.98 (0.75)	3.42 (0.64) *	3.38 (0.52) *	3.14 (0.61) *	2.79 (0.58) *	4.53 (0.83) ^
	PLA		4.10 (0.70)	3.39 (0.67) *	3.19 (0.68) *	2.95 (0.48) *	2.75 (0.53) *	4.45 (0.68) ^
Fat Ox (g/min)	BRJ		0.21 (0.15)	0.41 (0.14) *	0.41 (0.10) *	0.50 (0.15) *	0.67 (0.14) *	0.25 (0.18)
	PLA		0.15 (0.13)	0.39 (0.16) *	0.47 (0.21) *	0.55 (0.10) *	0.64 (0.12) *	0.21 (0.15)
RER	BRJ		0.96 (0.03)	0.93 (0.03) *	0.93 (0.02) *	0.91 (0.03) *	0.88 (0.03) *	0.96 (0.04)
	PLA		0.97 (0.02)	0.93 (0.03) *	0.91 (0.03) *	0.90 (0.02) *	0.89 (0.02) *	0.96 (0.02)
RPE	BRJ		11.1 (1.6)	11.7 (1.2)	12.4 (0.9) *	13.2 (1.1) *		15.8 (1.3) ^
	PLA		11.0 (1.8)	11.6 (1.3)	12.5 (1.0) *	13.1 (1.1) *		15.6 (1.4) ^
Heart Rate (bpm)	BRJ		141 (8)	154 (8) *	154 (7) *	156 (8) *	162 (9) *	172 (8) ^
	PLA		143 (7)	154 (9) *	154 (10) *	156 (9) *	158 (9) *	167 (8) ^
Lactate (mmol/L)	BRJ	2.4 (0.9) *	3.2 (0.8)	2.0 (0.9) *	2.1 (1.3) *	1.5 (0.4) *		3.7 (1.7)
	PLA	2.2 (0.7) *	2.9 (0.9)	1.8 (0.4) *	1.8 (0.8) *	1.6 (0.6) *		3.1 (0.8)

Data are mean (standard deviation) in crossover designed study with significance set at *p* < 0.05. * Different to 1 km. ^ Different to all other timepoints. CHO: carbohydrate, Ox: oxidation; RER: respiratory exchange ratio; RPE; rating of perceived exertion.

**Table 4:** Daily dietary intakes for phases of Study Two (Carb Max) in 19 elite male race walkers who followed interventions involving high CHO availability (MAX) or moderate CHO availability (CON).

		Pre Trial A	Pre Trial B		14 d Intervention		Pre Trial C	
		MAX (*n* = 9)	CON (*n* = 10)	MAX (*n* = 9)	CON (*n* = 10)	MAX (*n* = 9)	CON (*n* = 10)	MAX (*n* = 9)
Energy	kJ	16,262 (2234)	15,254 (2421)	15,150 (766)	15,183 (1786)	16,018 (950)	16,065 (2688)	16,474 (1254)
	kJ/kg	238 (26)	222 (28)	223 (10)	222 (7)	234 (7) *	232 (18)	242 (13)
CHO	g	782 (130) ^#$^^	526 (74)	534 (30)	454 (56)	694 (49) *	554 (82)	778 (104) ^#$^^
	g/kg	11.4 (1.6) ^#$^^	7.7 (0.9)	7.9 (0.3)	6.6 (0.2)	10.1 (0.3) *	8.0 (0.4)	11.4 (1.3) ^#$^^
Protein	g	101 (12) ^#$^^	133 (19)	133 (9)	147 (17)	135 (8)	140 (20)	108 (11) ^#$^^
	g/kg	1.5 (0.1) ^#$^^	1.8 (0.3)	2.0 (0.1)	2.1 (0.1)	2.0 (0.0)	2.0 (0.3)	1.6 (0.2) ^#$^^
Fat	g	37 (5) ^#$^^	102 (23)	96 (6)	127 (15)	49 (6) *	108 (26)	42 (15) ^#$^^
	g/kg	0.5 (0.1) ^#$^^	1.5 (0.3)	1.4 (0.1)	1.9 (0.1)	0.7 (0.1) *	1.6 (0.3)	0.6 (0.2) ^#$^^
Fibre	g	25.9 (5.4) ^#$^^	43.7 (7.0)	42.5 (3.3)	45.0 (4.9)	47.9 (2.4)	45.7 (9.0)	25.8 (5.8) ^#$^^

Daily intake presented as mean (SD) with significance noted at *p* < 0.05. * Different to CON Intervention; ^#^ Different to CON Trial B; ^$^ Different to MAX Trial B; ^^^ Different to CON Trial C.

**Table 5:** Data from a 26-km race walking protocol following Carb Max (MAX) or Control (CON)intervention in 19 elite male race walkers.

		CON (*n* = 10)							MAX (*n* = 9)						
Km		Pre	1	7	13	19	25	26	Pre	1	7	13	19	25	26
$\dot{V}$**O_2_** mL/kg/ min	Trial A									48.8 ^$^ (4.1)	48.5 ^$^ (3.9)	48.7 ^$^ (3.8)	49.4 ^$^ (3.3)	49.8 ^$^ (3.4)	56.6 (3.5)
	B		46.4 ^$^ (3.7)	46.0 ^$^ (4.0)	46.5 ^$^ (3.8)	46.5 ^$^ (3.7)	47.9 ^$^ (3.3)	53.3 (3.6)		48.7 ^$^ (4.2)	48.1 ^$^ (3.7)	48.5 ^$^ (4.0)	48.5 ^$^ (2.8)	48.7 ^$^ (3.6)	54.9 (3.8)
	C		46.6 ^$^ (3.7)	45.7 ^$^ (3.2)	46.1 ^$^ (3.4)	46.7 ^$^ (3.5)	47.5 (3.6)	53.8 (3.6)		49.3 ^$^ (3.6)	48.2 ^$^ (2.8)	48.5 ^$^ (3.7)	48.1 ^$^ (3.2)	48.7 ^$^ (3.4)	56.5 (4.1)
**RER**	A									0.98 (0.03)	0.96 ^$^ (0.02)	0.95 ^$^ (0.02)	0.94 ^$^ (0.03)	0.93 ^$^ (0.02)	1.00 (0.02)
	B		0.97 (0.05)	0.93 ^$^ (0.03)	0.92 ^$^ (0.03)	0.90 ^$^ (0.02)	0.88 ^$^ (0.04)	0.97 (0.03)		0.97 (0.03)	0.92 ^$^ (0.03)	0.92 ^$^ (0.03)	0.90 ^$^ (0.01)	0.88 ^$^ (0.01)	0.97 (0.02)
	C		0.96 (0.04)	0.92 ^$^ (0.03)	0.91 ^$^ (0.03)	0.90 ^$^ (0.02)	0.87 ^$^ (0.04)	0.95 (0.05)		0.96 (0.04)	0.94 ^$^ (0.03)	0.94 ^$^ (0.03)	0.94 ^$^^ (0.03)	0.93 ^$^^ (0.03)	0.98 (0.02)
**Heart Rate** (bpm)	A									146 ^$^ (12)	152 ^$^ (10)	155 ^$^ (7)	155 ^$^ (8)	159 ^$^ (9)	171 (9)
	B		144 ^$^ (12)	153 ^$^ (11)	155 ^$^ (10)	157 ^$^ (10)	163 ^$^ (9)	174 (8)		144 ^$^ (9)	154 ^$^ (8)	153 ^$^ (9)	155 ^$^ (8)	157 ^$^ (8)	169 (8)
	C #^		151 ^$^ (18)	148 ^$^ (11)	148 ^$^ (10)	150 ^$^ (10)	155 ^$^ (10)	166 (9)		138 ^$^ (10)	145 ^$^ (6)	144 ^$^ (5)	145 ^$^ (6)	149 ^$^ (8)	163 (5)
**RPE**	A									10.6 ^$^ (1.5)	11.4 ^$^ (1.0)	12.4 ^$^ (1.7)	13.2 ^$^ (1.8)		14.7 (1.8)
	B		11.8 ^$^ (1.5)	12.1 ^$^ (1.1)	12.9 ^$^ (1.2)	13.4 ^$^ (1.5)		16.7 (2.1)		10.9 ^$^ (2.0)	11.2 ^$^ (1.5)	12.2 ^$^ (1.2)	12.9 ^$^ (0.9)		15.2 (1.3)
	C		11.9 ^$^ (1.1)	12.2 ^$^ (0.9)	12.7 ^$^ (1.2)	13.7 ^$^ (1.3)		15.9 (1.1)		11.4 ^$^ (1.0)	11.9 ^$^ (0.9)	12.6 ^$^ (0.9)	13.1 ^$^ (0.9)		15.4 (0.7)
**Serum glucose** mmol/L	A								4.4 (0.8)	5.5 ^$^ (0.5)	6.9 * (0.5)	6.8 * (1.0)	7.2 * (0.8)		6.6 * (1.2)
	B	4.5 (0.7)	4.5 ^$^ (0.8)	6.4 * (0.9)	5.7 * (0.7)	5.5 * (0.7)		5.8 * (0.8)	4.4 (0.6)	4.3 ^$^ (0.6)	5.7 * (0.6)	5.3 * (0.7)	5.5 * (0.6)		5.8 * (0.6)
	C#	3.6 (0.4)	4.2 ^$^ (0.7)	5.8 * (1.1)	5.4 * (1.0)	5.0 * (0.5)		5.9 * (1.8)	3.7 (0.5)	4.4 ^$^ (0.6)	5.7 * (1.2)	5.9 * (1.6)	6.2 * (1.3)		6.2 * (0.9)
**Blood lactate** mmol/L	A								2.5 (0.8)	3.0 (0.9)	1.9 ^$^ (0.6)	2.4 ^$^ (0.8)	1.7 ^$^ (0.4)		4.0 * (1.5)
	B	2.3 (0.6)	3.3 (1.1)	1.9 ^$^ (0.7)	1.8 ^$^ (0.8)	1.6 ^$^ (0.4)		3.6 (0.9)	2.1 (0.8)	2.8 (1.2)	1.6 ^$^ (0.5)	1.7 ^$^ (0.6)	1.9 ^$^ (1.5)		2.7 * (0.7)
	C	2.5 (0.7)	3.0 (0.8)	2.0 ^$^ (0.6)	1.9 ^$^ (0.8)	2.0 ^$^ (0.9)		2.9 (1.0)	2.9 (0.8)	3.5 (0.9)	2.4 ^$^ (1.1)	1.8 ^$^ (0.5)	2.6 ^$^ (1.3)		3.9 * (1.8)

Data are mean (standard deviation) with significance set at *p* < 0.05. * Significantly different to Pre-ex. ^$^ Significantly different to 26 km. ^#^ Significant main effect for within group differences compared to Trial A. ^ Significant main effect for within group differences compared to Trial B. $\dot{V}$O_2peak_: peak aerobic power; RER: respiratory exchange ratio; RPE; rating of perceived exertion.

## Data Availability

The data presented in this study are available on request from the corresponding author.
